# Scintigraphic evaluation of the osteoblastic activity of rabbit tibial defects after HYAFF11 membrane application

**DOI:** 10.1186/s13018-016-0393-y

**Published:** 2016-05-03

**Authors:** Musa Uğur Mermerkaya, Mahmut Nedim Doral, Fatih Karaaslan, Gazi Huri, Seyhan Karacavuş, Burak Kaymaz, Erkan Alkan

**Affiliations:** Department of Orthopaedics and Traumatology, Medical School, Bozok University, Yozgat, Turkey; Department of Orthopaedics and Traumatology, Medical School, Hacettepe University, Ankara, Turkey; Department of Nuclear Medicine, Medical School, Bozok University, Yozgat, Turkey; Department of Orthopaedics and Traumatology, Medical School, Çanakkale Onsekiz Mart University, Çanakkale, Turkey; Department of Orthopaedics and Traumatology, Yalvaç State Hospital, Isparta, Turkey

**Keywords:** Hyaluronan-based mesh, Osteoblastic activity, Bone healing, Bone scintigraphy

## Abstract

**Background:**

An unfavorable condition for bone healing is the presence of bone defects. Under such conditions, a material can play a role to cover fractured or defective bone. Technological advances now allow for the use of such material. Hyalonect^®^ (Fidia Advanced Biopolymers SLR, Italy), a novel membrane comprising knitted fibers of esterified hyaluronan (HYAFF11) can be used to cover fractured or grafted bone and can also serve as a scaffold to keep osteoprogenitor cells in place. The aim of this study was to compare osteoblastic activity by the use of scintigraphic methods in defective rabbit tibias during early-phase bone healing with or without a hyaluronan-based mesh.

**Methods:**

Two groups (A and B) of New Zealand albino rabbits were used; each group included 10 animals. Operations on all rabbits were performed under general anesthesia. We also resected 10-mm bone segments from each animal’s tibial diaphysis. After resection, tibias with defects were fixed using Kirschner wires. In group A, no hyaluronan-based mesh was used. In group B, tibial segmental defects were enclosed with a hyaluronan-based mesh. The rabbits were followed up for 4 weeks postoperatively, after which bone scintigraphic studies were performed on each animal to detect and compare osteoblastic activity.

**Results:**

The mean count in the fracture side of the hyaluronan-based mesh group was significantly higher compared to that of the group A (*p* = 0.019). However, there was no significant difference between group B and control rabbits with respect to the mean count on the intact bone side (*p* = 0.437). The bone defect (fracture)/intact bone mean count ratio was significantly higher in group B compared to group A (*p* = 0.008).

**Conclusions:**

A hyaluronan-based mesh plays a role in promoting osteoblastic activity. Hyalonect^®^ is suitable for restoring tissue continuity whenever the periosteal membrane is structurally impaired or inadequate. Our results demonstrated that, during early-phase bone healing, osteoblastic activity was increased in bone defect sites when a hyaluronan-based mesh was also used. The most important aspect of this study concerns its scintigraphy-based design. This study is the first to use a scintigraphic method to demonstrate the effectiveness of hyaluronic acid-based material for bone healing.

## Background

During trauma or surgery, disruption in the periosteum can occur at the fracture site. The periosteum provides external blood supply to the bone and plays an important role in healing. Given its importance to bone vitality, maximum attention must be directed toward the preservation of the periosteum during fracture surgery [1]. An impaired periosteum can cause deterioration in the biological environment of the bone, which may affect patients’ treatment results and quality of life negatively, even when an appropriate fixation technique is used. The presence of bone defects represents another unfavorable condition for bone healing. Callus formation and the maintenance of bone integrity are significantly more difficult with larger defects, which are frequently filled using autografts or allografts [2]. Allografts are predominantly osteoconductive, whereas autografts are, to a certain extent, both osteoconductive and osteoinductive [3, 4]. For allografts in particular, the presence of osteoprogenitor or mesenchymal stem cells must precede the fracture site and then be maintained to achieve healing; this process is more difficult when the periosteum is impaired. Under such conditions, a material can play a role to cover fractured or defective bone. Innovations and technological advances have rendered this possible through the use of a hyaluronan-based mesh, which can be used to cover fractured or grafted bone and can also provide a scaffold to keep osteoprogenitor cells in place [5–7]. Hyalonect^®^, a bio-resorbable knitted mesh used during orthopedic trauma and reconstructive surgery, is composed of HYAFF^®^, a benzyl ester of hyaluronic acid (HA) and natural component of the extracellular matrix. The mesh is resorbable and may be fixed to the surgical site by applying sutures or internal bone fixation devices.

Bone scintigraphy, a radionuclide bone metabolism imaging technique that is highly sensitive to osteoblastic activity in the skeleton, can measure the distribution of a radiolabelled phosphorous compound (^99m^Tc-MDP) around the defect site [8]. The technique is dependent on bone metabolism rate and blood flow [9]. The aim of this study was to compare osteoblastic activity in defective rabbit tibias during early-phase bone healing, using a scintigraphic method with or without a hyaluronan-based mesh.

## Methods

Twenty adult male New Zealand white rabbits, aged 12 months and weighing 4000–4500 g, were included in the study. The study protocol was approved by the local Ethics Committee (Çukurova Medical School No. 7; 30 October 2014). The rabbits received care in accordance with the guidelines of the Ethics Committee pertaining to the use of animals in experimental studies. We randomly assigned the rabbits into two groups (A and B; both *n* = 10).

### Surgical procedure

**Fig. 1:**
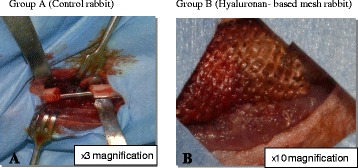
**a** Intraoperative image in group A (control group). **b** Intraoperative image in group B (hyaluronan-based mesh group)

First, 22-G catheters were placed in the lateral auricular vein of the rabbits, who were anesthetized by injecting 10 mg/kg of propofol. During surgery, the 10 mg/ml propofol solution was infused at a rate of 30 ml per hour as necessary. A tibial diaphysial segmental bone defect approximately 10 mm in diameter was created using an oscillating saw blade. Saline irrigation was used to prevent thermal bone necrosis. Hyaluronan-based mesh replacement was performed in accordance with the study design. After resection, tibias with defects were fixed using Kirschner wires (K-wires). In group A, no hyaluronan-based mesh was used (Fig. 1a), whereas tibial segmental defects were enclosed with a hyaluronan-based mesh in group B (Fig. 1b). Iliac wings of the rabbits were prepared for all rabbits, and blood was aspirated from the iliac wings which provided a source of mesenchymal stem cells for stimulating and accelerating bone healing process. In both groups, blood aspirated from the iliac wings of the rabbits was injected (2 cm^3^) into the tibial defects. Surgery was then completed with suturing and dressing of the surgical wounds. Long leg castings were then performed. The animals were examined regularly everyday to assess complications and wound healing using a cast window; all received the same amount and quality of food.

During the 2-week follow-up period, one rabbit in each group died due to wound complications. After 2 weeks, long leg casts were removed. The remaining 18 rabbits were followed up for 4 weeks postoperatively. At the end of this period, bone scintigraphy was performed on each rabbit to assess osteoblastic activity.

### Scintigraphic study

**Fig. 2:**
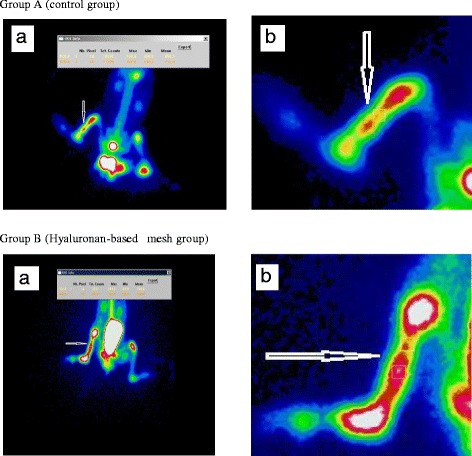
**a** (*a*) Radionuclide imaging in group A (control group). (*b*) The *black arrow* shows the zoom image of the indicator of the ROI in group A (control group). **b** (*a*) Radionuclide imaging in group B (hyaluronan-based mesh group). (*b*) The *black arrow* shows the zoom image of the indicator of the ROI in group B (hyaluronan-based mesh group)

The operations were performed using radionuclide bone imaging in both groups at 4 weeks postoperatively. During scanning, the animals were under ketamine hydrochloride anesthesia; they were placed in a supine position on the scanner bed, with their hind paws stretched and fixed in a custom-made wooden holder. Each animal was given an intravenous injection of 18.5 MBq/kg bolus technetium-99m methylene diphosphonate (^99m^Tc-MDP; Eczacibasi Monrol, Kocaeli, Turkey). Three-phase radionuclide imaging was started using a gamma camera (Philips Medical Systems Brightview, Best, Holland) equipped with a low-energy, high-resolution, pinhole collimator set at 140 KeV with a 20 % window and 64 × 64 matrix size, immediately after injection for 2 min. Static images were obtained 4 h after injection of a radiotracer in a 256 × 256 matrix. We analyzed images to identify regions of interest (ROIs) on the intact and fractured sides (Fig. 2). All ROIs were identical in size and shape. Total radioactivity counts in each ROI and the intake ratio (average counts on the fractured side/average counts at the intact side) were derived and compared between the groups.

### Statistical analysis

#### Sample size estimation

The primary aim of this study was to compare differences in the arithmetic mean ratio (i.e., bone defect [(fracture)/bone] between the control and hyaluronan-based mesh groups. A total sample size of 16 (*n* = 8 for each group) was required to detect an at least 0.11 difference between the groups with a power of 80 % at the 5 % significance level. This difference was taken from both the pilot study and our clinical experiments. Sample size estimation was performed using the G*Power software package (ver. 3.0.10; Kiel, Germany).

Data analysis was performed using the SPSS for Windows software package (ver. 11.5; SPSS Inc., Chicago, IL, USA). The normality of the distribution of continuous variables was determined using Shapiro-Wilk test. Data are provided as means ± SD. Group differences in clinical measurements (i.e., scintigraphic counts on fracture and normal bone and ratio of these values) were assessed using Student’s *t* test. A *p* value <0.05 was taken to indicate statistical significance.

## Results

The mean count/pixel ratio on the bone defect (fracture) side in the hyaluronan-based mesh group was significantly higher compared to that in the group A (*p* = 0.019). However, there was no significant difference between the hyaluronan-based mesh and control rabbits in mean count/pixel ratio on the intact bone side (*p* = 0.437; Table 1).

**Table 1:** Scintigraphy counts in group A and group B

Variable	Group A (*n* = 9)	Group B (*n* = 9)	*p* value*
Bone defect (fracture)	2768.6 ± 257.34	3203.7 ± 428.15	*0.019*
Bone	1649.2 ± 101.36	1688.2 ± 106.30	0.437
Ratio	1.68 ± 0.095	1.89 ± 0.188	*0.008*

Values are given as means ± SD

*Student’s *t* test. *p* value <0.05 was taken to indicate statistical significance

The bone defect (fracture)/bone mean count/pixel ratio was significantly higher in group B compared to group A (*p* = 0.008; Table 1).

## Discussion

The major finding of this study was during early-phase bone healing, osteoblastic activity is increased in the defect site due to more rapid bone metabolism when a hyaluronan-based mesh is also used.

Commensurate with recent biotechnological advances, surgeons now aim to replace tissues that are injured, are irreparable, or possess low regeneration potential with biosynthetic materials possessing the same function or structure. One such biosynthetic material is Hyalonect^®^, which has been used previously for chondral lesions and also has the potential to participate in periosteal regeneration due to its hyaluronate structure, which plays a role in extracellular matrix formation, angiogenesis, and osteogenesis [7, 10, 11]. Rhodes et al. [5] reported that Hyalonect^®^ is capable of restoring the function of damaged connective tissues such as the periosteum without interfering with the natural tissue repair process.

Numerous imaging modalities are used to assess fracture healing. Traditionally, plain radiographs have been employed to serially follow callus formation. The theoretical basis for the use of ^99m^Tc-labeled phosphates in the diagnosis of bone pathology is based on their high affinity for hydroxyapatite and immature collagen. ^99m^Tc-MDP is adsorbed selectively onto the mineral phase of forming bone (via hydroxyapatite crystals) [8]. The accumulation of technetium-99m-labeled diphosphonates is remarkably consistent with the amount of mineralization caused by the activity of osteoblastic cells following the onset of calcification. An approximately 10 % increase in osteoblastic activity, above normal levels, can be detected by scintigraphic studies but cannot be seen on conventional radiographs [8]. Unlike radiographic and histologic methods, bone scintigraphic images can reveal early bone mineralization in the skeleton during the healing process [12, 13]. Bone scintigraphy is a feasible, easily applied, reproducible method for objective assessment of bone healing [8].

In this study, we demonstrated the periosteum-like activity of this biomaterial and its role in promoting osteoblastic activity. The most important aspect of this study lies in its scintigraphy-based design. The results indicate that, during early-phase bone healing, osteoblastic activity is increased in the defect site due to more rapid bone metabolism when a hyaluronan-based mesh is also used. The hyaluronan-based mesh group exhibited the highest count/pixel ratio, for coated hyaluronan-based mesh sites, indicative of extensive osteoblastic activity and increased metabolism.

Several studies have demonstrated osteoblastic activity-promoting effects of a hyaluronan-based mesh. In one such preclinical study, conducted by Rhodes et al., holes were drilled in the humerus of the canines [5]. The holes were then filled with bone graft materials and surrounded with a hyaluronan-based mesh. Histologic evaluation demonstrated the effect of the hyaluronan-based mesh on healing; the material surrounded the tissues, and a greater number of host cells and reduced fibrous tissue formation were evident.

In another study, Aslan et al. formed bone cavities in the tibias of rabbits [14]. In one group, the cavities were filled only with spongiosal bone graft. In another group, they were filled with a viscoelastic preparation of hyaluronic acid in addition to spongiosal bone grafts. The hyaluronic acid group had higher histopathologic scores compared to the graft-only group. Ayanoğlu et al. showed that Hyalonect^®^ and grafting significantly enhance the healing process when used alone or together. The use of both Hyalonect^®^ and grafting together resulted in better early radiological healing than bone grafting alone [15]. Tekin et al. reported that Hyalonect^®^ use during surgical treatment of long-bone non-infected pseudoarthrosis is a safe method that promotes bone union [6]. In their study, a pseudoarthrosis site was fixated internally and filled with allografts surrounded with Hyalonect^®^; union was achieved in all patients after approximately 6 months. The findings from our studies are consistent with those cited in previously published studies. The results of our study demonstrated that Hyalonect^®^ significantly increases the osteoblastic activity.

The main limitation of the study is the experimental study performed on tibial diaphysial defect of rabbits, and hence, we cannot be sure of the same beneficial effect of a hyaluronan-based mesh on human bones unless we perform a clinical study. We also did not use other bone substitutes to compare the results. Further studies should be performed to compare Hyalonect^®^ with other bone substitutes. Another limitation of the study is that we also did not use biomechanic, radiographic, and histologic methods to compare the results.

Hyalonect^®^ is suitable for restoring tissue continuity whenever the periosteal membrane is structurally impaired or inadequate and plays a role in promoting osteoblastic activity. This study demonstrated that, during early-phase bone healing, osteoblastic activity was increased in bone defect sites when a hyaluronan-based mesh was also used.

## Conclusions

Hyaluronic acid is an important extracellular matrix element due to its effects on processes such as cellular vitality, adhesion, interaction, migration, osteoblastic activity, and tissue repair [16–22]. This study is the first to use a scintigraphic method to demonstrate the effectiveness of a hyaluronic acid-based material for bone healing. A hyaluronan-based mesh plays a role in promoting osteoblastic activity. Hyalonect^®^ is suitable for restoring tissue continuity whenever the periosteal membrane is structurally impaired or inadequate. By fulfilling these functions, artificial hyaluronic acid-based materials cover bare bone or bone grafts, thereby facilitating the treatment of fractures, non-unions, or pseudoarthrosis in a safer and more effective manner. A hyaluronan-based mesh can be used in such cases.

### Future

In the near future, innovations will contribute greatly toward tissue engineering in the area of orthopedics, particularly with respect to the cartilage and periosteum. Significantly improved comfort and easier healing will be possible, and the success rate of orthopedic treatment will improve.
